# Influence of an e-mail with a drug information attachment on sales of prescribed drugs: a randomized controlled study

**DOI:** 10.1186/1472-6904-7-12

**Published:** 2007-10-18

**Authors:** Christina Edward, Anders Himmelmann, Susanna M Wallerstedt

**Affiliations:** 1 Department of Health, Pharmaceutical Unit, Regionens hus, SE-405 44 Göteborg, Sweden; 2 Department of Clinical Pharmacology, Sahlgrenska University Hospital, SE-413 45 Göteborg, Sweden

## Abstract

**Background:**

To provide doctors with producer-independent information to facilitate choice of treatment is an important task. The objective of the present study was to evaluate if an e-mail with a drug information attachment has effects on sales of prescribed drugs and if the design of the attachment is of importance.

**Methods:**

The Swedish pharmaceutical benefit board found rizatriptan (Maxalt^®^) 10 mg to be the most cost-effective triptan. All 119 heads of primary care units in western Sweden were randomized to receive information concerning this conclusion via (i) e-mail with attachment I, (ii) e-mail with attachment II or (iii) no information (control). Attachment I was a short one (heading plus three lines text), whereas attachment II was a long one (heading plus one page text and one page with tables). The change in percentage rizatriptan of total triptans sold before and after the intervention (May – July 2004 and May – July 2005, respectively) was compared between the groups.

**Results:**

Totally 48,229 (2004) and 50,674 (2005) defined daily doses of triptans were prescribed and sold during May – July in primary care units in the western part of Sweden. The absolute change in percentage rizatriptan was greater in the intervention groups compared with the control group 2 (25^th ^– 75^th ^percentile: -3 – 7) vs 0 (-7 - 5), P = 0.031). The absolute change in percentage rizatriptan did not differ between the two attachment groups (P = 0.93).

**Conclusion:**

An e-mail with a drug information attachment may influence sales of prescribed drugs. No difference between different designs of the attachment could be detected.

## Background

Drugs are keystones in the treatment of patients. Rational treatment with drugs requires adequate knowledge on drugs' benefits, risks and cost-effectiveness. The increasing volume of information related to drugs and prescribing may make it difficult for an individual primary care doctor to keep up to date with best practice. Lack of time for reading and evaluating scientific papers in favour of direct patient work may be one explanation. To consider costs when prescribing may add an additional difficulty. However, in a survey, the majority of the doctors stated that costs of medicines are important and they were willing to make economic considerations in prescribing [1]. Therefore, it seems reasonable to provide doctors with producer-independent information including cost-effectiveness to facilitate choice of treatment.

The results of research and development have to be conveyed to practicing doctors. Producer-independent information on drugs can be provided in different ways. Oral information combined with reminding notes has been shown to influence prescription patterns [2] as well as interactive teleconference [3]. Cochrane reviews indicate that audit and feedback [4] as well as interactive workshops [5] and educational outreach visits [6] can be effective in improving professional practice. However, these methods are time-consuming and expensive. Other supplementary methods to convey new knowledge are needed.

In the western part of Sweden, news concerning drugs is distributed to the heads of each primary care unit via attachments in e-mails from the regional department of health, pharmaceutical unit, with a standard format of the attachments. The head is instructed to forward the message to the employees. To the best of our knowledge, no reports on effects of e-mails with drug information on drug sales have been published.

The aims of the present study were to evaluate if these e-mails with drug information attachments have effects on sales of prescribed drugs and if the design of the attachment is of importance.

## Methods

In Sweden, the pharmaceutical benefits board (Läkemedelsförmånsnämnden, LFN) decides if a pharmaceutical product is to be included in the pharmaceutical benefits scheme and be reimbursed by the state. Amongst other things, the decision is based on cost-effectiveness. In 2004, an evaluation of triptans, used for migraine treatment, was presented. Rizatriptan (Maxalt^®^) 10 mg was found to be the most cost-effective triptan [7].

Public primary care units in the western Sweden are directed by totally 119 heads, all of which were included in the present study. In order to compare the effects of two drug information alternatives on percentage rizatriptan sales in an unbiased fashion, the allocation was by randomization. Each head of a primary care unit constituted a randomization unit and these were arranged in a list, geographically related units being placed together. A 1:1:1 allocation sequence consisting of randomized permuted blocks was then applied to this list on the 28^th ^of April 2005, i.e. before the e-mails were sent. A person not involved in the study and without knowledge about the study protocol performed the randomization procedure.

The three randomization groups received (i) an e-mail with attachment I, (ii) an e-mail with attachment II or (iii) no e-mail at all. The attachments followed the standard settings of e-mails from the regional department of health, pharmaceutical unit. Attachment I was a short message consisting of the heading 'Prescribe Maxalt^® ^10 mg when a triptan is needed for migraine treatment', followed by three lines of text. Attachment II was a long message consisting of the same heading as attachment I, followed by one page of text and one page with three tables. The name of the attachment was identical to other drug information attachments from the regional department of health, pharmaceutical unit. The attachments can be supplied from the corresponding author by request. The e-mails were sent the last work day of April 2005. The subject of the e-mail was 'to be forwarded to the prescribers' and in the e-mail the following text was included: 'Information on the LFN review of triptans. To be forwarded to the prescribers in the primary care unit'. The primary care units were not informed about the study.

Apoteket AB has monopoly of prescription drug sales in Sweden. A national prescription register (Xplain) was established in the late 1990s to improve possibilities for drug utilization studies. Data on age, sex and residential area of the patient, as well as information on the prescriber and the drug dispensed (e.g. number of defined daily doses (DDD) and costs) are routinely gathered when prescriptions are dispensed at Swedish pharmacies. Xplain was used for evaluation of effects on sales of prescribed drugs by the e-mail.

### Statistics

The percentage rizatriptan of total triptans sold (DDD) was calculated for each unit in the randomization groups. By use of Mantel's test [8], comparisons of absolute change of percentage rizatriptan before and after the intervention (May – July 2004 and May – July 2005, respectively) between the groups were performed. In this case Mantel's test meant that the comparisons were performed within subgroups of units all of which belonged to the same interval of baseline proportion. The results of the subgroups were pooled to one test for each comparison. By that technique the influence of the baseline proportion was eliminated. Values are presented as median (25^th ^– 75^th ^percentile). A P-value < 0.05 was considered significant.

## Results

**Figure 1:**
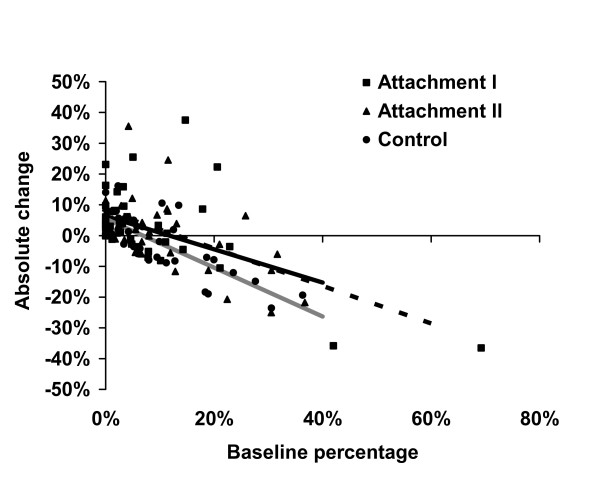
**Absolute change in percentage rizatriptan (after intervention minus baseline) depending on randomization group and baseline percentage**. Regression lines are indicated in the figure, broken black line denotes attachment I, solid black line denotes attachment II and solid grey line denotes control.

Totally 48,229 (2004) and 50,674 (2005) DDD triptans were sold during May – July in primary care units in the western part of Sweden. Baseline sold triptans in the primary care units in the three randomization groups was (DDD): 377 (244 – 544) (attachment I, n = 40), 333 (197 – 592) (attachment II, n = 40) and 386 (200 – 509) (control, n = 39). Percentage rizatriptan at baseline and absolute change in percentage rizatriptan after the intervention in the randomization groups are illustrated in Figure 1.

Percentage rizatriptan before and after the intervention as well as absolute and relative change is described in Table 1. The absolute change in percentage rizatriptan was greater in the combined intervention groups compared with the control group [2 (-3 – 7) vs 0 (-7 - 5), P = 0.031]. The absolute change in percentage rizatriptan did not differ between the two attachments (P = 0.93).

**Table 1:** Percentage rizatriptan of prescribed and sold triptans before (May – July 2004) and after (May – July 2005) the intervention and absolute change in percentage rizatriptan (after minus before).

	Percentage rizatriptan before the intervention	Percentage rizatriptan after the intervention	Absolute change	Relative change	P-value
Control	6 (2 – 13)	7 (2 – 11)	0 (-7 - 5)	-29 (-69 – 83)	-
Attachment I	3 (1 – 10)	8 (3 – 14)	3 (-2 – 8)	42 (-51 – 222)	0.071
Attachment II	7 (1 – 13)	8 (2 – 17)	1 (-4 – 6)	-1 (-59 – 70)	0.052
Attachment I + II	5 (1 – 12)	8 (3 – 16)	2 (-3 – 7)	20 (-54 – 140)	0.031

Mantel's test was used for comparison of absolute change vs control. Values are presented as median percentage (25^th ^– 75^th ^percentile).

## Discussion

The results of research and development have to be conveyed to practicing doctors. Generally, drug information to primary health care doctors is given by pharmaceutical sales representatives. It has been shown that frequent visits by representatives are associated with increased prescribing costs [9,10]. To the best of our knowledge, no information on effects on quality of prescription is available. Producer-independent information may have an advantage in this aspect, since the primary aim is not to sell drugs, but rather to improve health care.

The results of the present study indicate that producer-independent drug information via an e-mail attachment may have a modest effect on sales of prescribed drugs. No difference between different designs of the attachment could be detected. Thus, e-mails with attachments may be an alternative or a complement to other interventions, e.g. education. The difference between prescribing and sales may be substantial since many prescriptions are not dispensed. Hence, the results of the present study cannot directly be interpreted as effects on prescription patterns.

In the present study, we do not know to what extent the e-mail actually reached the target population, the prescribing doctors, since it was only administered to the heads of the primary care units. If the forwarding process was not adequate, the effect of a drug information attachment in an e-mail on prescription patterns may have been underestimated. Moreover, the information in the attachment may also have reached the prescribing doctors in the control group. These factors may have influenced the results.

One problem for doctors is the large amount of information available and the sparse amount of time to assimilate the information. The present study does not increase the doctors' time for reading, but indicates that doctors may have time to read an e-mail with an attachment from a trustworthy source and this may have an effect on the prescription.

The present study only allows conclusions regarding effects on sales of prescribed drugs during the three months follow-up. The effects of other interventions on prescription patterns seem to diminish three to four months after the intervention [2,11]. One advantage with the 'e-mail attachment intervention' is that it can be repeated without being costly. However, the results of the present study only apply to one single message. The effects of multiple e-mails or repeated e-mails need to be further explored.

## Conclusion

The present study indicates that an e-mail with a drug information attachment may influence sales of prescribed drugs. No difference between different designs of the attachment could be detected. E-mail with attachment may thus be an alternative to provide doctors with producer-independent drug information.

## Competing interests

The author(s) declare that they have no competing interests.

## Authors' contributions

CE participated in the design of the study, carried out the acquisition of data and revised the manuscript. AH conceived the study, participated in its design and revised the manuscript. SMW conceived the study, participated in its design and drafted the manuscript. All authors read and approved the final manuscript.

## Pre-publication history

The pre-publication history for this paper can be accessed here:


